# Supplementary material for: Endosymbiont hijacking of acylcarnitines regulates insect vector fecundity by suppressing the viability of stored sperm
Source: PLoS Genet. 2025 Dec 10;21(12):e1011974. doi: 10.1371/journal.pgen.1011974 (PMC12707635; doi:10.1371/journal.pgen.1011974)
Supplement: Attachment — Submitted filename: Weiss et al_REVIEW.pdf [file pgen.1011974.s017.pdf]

Weiss et al., in their manuscript “Endosymbiont hijacking of acylcarnitines regulate insect vector fecundity by suppressing the viability of stored sperm” the authors, using a series of elegant experiments, provide evidence consistent with their hypothesis that the bacterial symbiont Spiroplasma compete with spermatozoa stored in female tsetse fly spermathecae for acylcarnitines produced by the host female.

Most of the experimental work was completed in laboratory reared *Glossina fuscipes fuscipes* (*Gff*) using the bacterium *Spiroplasma glossinidia* (*sGff*). The symbiont *sGff* is maternally transmitted, infecting tsetse gut, reproductive tracts, and hemolymph. *sGff* infected tsetse are more resistant to trypanosome infection and have reduced fecundity relative to uninfected individuals. Some work was also completed in field collected *Gff*, and some work was completed in laboratory reared *Glossina mortisans mortisans* because it does not contain *Spiroplasma*.

The authors convincingly show the following:

1) Sperm motility is not affected by Spiroplasma infection status in the male reproductive tract
2) Sperm motility is lower in the spermathecae of Spiroplasma infected females relative to uninfected females
3) The abundance profile of acylcarnitines in differs between Spiroplasma infected and uninfected females, with some acylcarnitines being significantly depleted in infected versus uninfected females.
4) Many of the experiments focus on *Gffcpt1* a gene encoding carnitine O-palmitoyltransferase-1, an enzymatic component of the carnitine shuttle that couples fatty acyl-CoAs to L-carnitine to form acylcarnitines – thus an enzyme essential to the production of acylcarnitines.
5) Knockdown of *Gffcpt1* expression reduces sGff density
6) *Gffcpt1* is turned on in the spermathecae of infected and uninfected flies following mating, with laboratory, and field data from two locations, all showing that expression is higher in infected versus uninfected flies
7) *Gffcpt1* knockdown flies do not show altered sperm motility relative to GFP controls in testes, but the motility of sperm in spermathecae is reduced in *Gffcpt1* knockdown flies relative to GFP controls.
8) The impact of *Gffcpt1* knockdown does not impact early reproduction but it does significantly reduce and eventually eliminate reproduction in the third and fourth GC (need to define GC – does it mean “gonotrophic cycle”??)

Take together the work is compelling.

I have a few comments/questions that will help improve the manuscript.

1) Include Table S1 in the manuscript. For those not familiar with the naming of acylcarnitines (probably most readers of this manuscript), the presence of the table in the main text would be very helpful.
2) Line 114: Define GC – what is this??
3) Line 177: FB = Fat Body. It seems unnecessary to abbreviate throughout
4) Line 199: "[shown here…" Where?? It was not clear where in the current study this is shown.
5) Lines 188-214 – I got really quite confused understanding where expression of acylcarnitines is up and where it is down, it seems there are differences in different tissues, but keeping this straight was impossible – a figure or table summarizing these patters would be very helpful
6) Line 240: "imbalance" – is there a different word that could be used here? How do you know that the different profile = an imbalance?
7) Line 325: "to provide" seems to be overly confident language … it seems that the authors are speculating that this is the reason
8) Figure 4, panel E (lines 389-390) – why is it only the section marked by the dotted line and not the tissue around the whole spermathecae – there does not seem to be a difference in the tissue in the marked section of the tissue versus the whole yellowish halo
9) Line 416; "the enzyme" – what enzyme?
10) Line 605: replace describing with described
11) Figure 5 – I did not find this figure to be at all helpful, it made no sense to me. It would be great to have a figure that does clearly explain the findings and the model of regulation/competition proposed by the authors, but this figure it is not it.
12) General comment on all figures, to work to increase the labelling on the figures to reduce the reader's dependence on the figure legends. Doing this will increase the readability and accessibility of the manuscript.

Overall, a cool story, supported by good work.
