# Supplementary material for: Endosymbiont hijacking of acylcarnitines regulates insect vector fecundity by suppressing the viability of stored sperm
Source: PLoS Genet. 2025 Dec 10;21(12):e1011974. doi: 10.1371/journal.pgen.1011974 (PMC12707635; doi:10.1371/journal.pgen.1011974)
Supplement: Attachment — Submitted filename: Weiss et al_Response to Reviewers.docx [file pgen.1011974.s019.docx]

21 November, 2025

Dear Dr. Vogels and PLoS Genetics Reviewers,

We would like to thank you for your comprehensive review of our manuscript, entitled ‘Endosymbiont hijacking of acylcarnitines regulates insect vector fecundity by suppressing the viability of stored sperm’. In the following pages we provide an italicized response to each of the reviewer comments, which are listed in the order they originally appeared. We also provide two versions of the revised manuscript, one with tracked changes and one ‘clean’ version.

Again, we thank the editor and reviewers for taking the time to read our manuscript and provide valuable feedback about our research. We feel that your comments and suggestions significantly improved the manuscript, which we hope is now suitable for publication in PLoS Genetics.

Sincerely,

Brian Weiss, Fabian Gstöttenmayer, Erick Awuoche, Gretchen Smallenberger, Geoffrey Attardo, Francesca Scolari, Robert Koch, Daniel Bruzzese, Richard Echodu, Robert Opiro, Anna Malacrida, Adly Abd-Alla, and Serap Aksoy

**Journal Requirements**:

If the reviewer comments include a recommendation to cite specific previously published works, please review and evaluate these publications to determine whether they are relevant and should be cited. There is no requirement to cite these works unless the editor has indicated otherwise.

1) Please ensure that the CRediT author contributions listed for every co-author are completed accurately and in full. At this stage, the following Authors/Authors require contributions: Brian L Weiss. Please ensure that the full contributions of each author are acknowledged in the "Add/Edit/Remove Authors" section of our submission form. The list of CRediT author contributions may be found here: <https://journals.plos.org/plosgenetics/s/authorship#loc-author-contributions>

*Brian Weiss’s CRediT author contributions to the study have been completed.*

2) We do not publish any copyright or trademark symbols that usually accompany proprietary names, eg ©,  ®, or TM  (e.g. next to drug or reagent names). Therefore please remove all instances of trademark/copyright symbols throughout the text, including:
- © on page: 30, line 779
- ® on page: 34, line 885
- TM on page: 34, line 885

*We have removed these three symbols from the revised manuscript.*

3) We have noticed that you have uploaded Supporting Information files, but you have not included a list of legends. Please add a full list of legends for your Supporting Information files after the references list.

*We have added the Supporting Information legends after the reference list.*

4) Some material included in your submission may be copyrighted. According to PLOSu2019s copyright policy, authors who use figures or other material (e.g., graphics, clipart, maps) from another author or copyright holder must demonstrate or obtain permission to publish this material under the Creative Commons Attribution 4.0 International (CC BY 4.0) License used by PLOS journals. Please closely review the details of PLOSu2019s copyright requirements here: PLOS Licenses and Copyright. If you need to request permissions from a copyright holder, you may use PLOS's Copyright Content Permission form.
Please respond directly to this email and provide any known details concerning your material's license terms and permissions required for reuse, even if you have not yet obtained copyright permissions or are unsure of your material's copyright compatibility. Once you have responded and addressed all other outstanding technical requirements, you may resubmit your manuscript within Editorial Manager. 
Potential Copyright Issues:
i) Figure 5. Please confirm whether you drew the images / clip-art within the figure panels by hand. If you did not draw the images, please provide (a) a link to the source of the images or icons and their license / terms of use; or (b) written permission from the copyright holder to publish the images or icons under our CC BY 4.0 license. Alternatively, you may replace the images with open source alternatives. See these open source resources you may use to replace images / clip-art:

*We generated this image (and S10 Fig.) ourselves, thus there should be no associated copyright issues.*

**Reviewer #1**:

The bases of most bacterial symbioses in insects are poorly understood mechanistically. This is a beautiful study exploring a possible cause of competition between spiroplasmas and the tsetse fly. The methods are clearly described and robust. The inclusion of field collected tsetse’s is a nice addition, even though they cannot be age controlled. The strength of the effects are strong and believable – sperm motility, increase in acylcarnitines despite overall reduction in lipids, reduction in spiroplasmas following RNAi of acylcarnitine synthesis gene, and patterns of gene expression that correlate with spiroplasma infection, mating, and beat frequency. The assembly of experimental approaches gives weight to the authorr's arguments. The discussion is thoughtful and incorporates relevant data across systems to provide support for speculative intepretation.

This paper was a joy to read!

Minor
Line 67 awkward. Reword.

*In the revised version of the manuscript (line 68) we have broken this sentence into two that now read as follows: Many female animals, prominent among them insects, store spermatozoa in specialized organs after copulation. Stored spermatozoa are subsequently used to fertilize eggs that ovulate during consecutive reproductive cycles.*

Line 92 ‘they’ refers to trypanosomes. Confusing. Reword.

*In the revised version of the manuscript (line 94) we have changed this sentence to read as follows: While only a small percentage of tsetse are infected with trypanosomes (~10-15%), these flies all house a population-dependent assortment of viruses and transient and maternally-transmitted endosymbiotic bacteria.*

Line 175. Remind reader why lipidomics rather than a metabolomics approach.

*Our lab has previously performed extensive research on the conspicuous physiological role that lipids play in supporting tsetse’s reproductive homeostasis. With that in mind, we have addressed the reviewer’s useful suggestion by changing this sentence (starting on line 180 in the revised manuscript) to read as follows: We chose to specifically identify and quantify the lipids produced by tsetse’s fat body (which is the primary source of lipids in the fly) because these molecules play a prominent and well characterized role in maintaining tsetse’s reproductive homeostasis.*

**Reviewer #2**:

Weiss et al., in their manuscript “Endosymbiont hijacking of acylcarnitines regulate insect vector fecundity by suppressing the viability of stored sperm” the authors, using a series of elegant experiments, provide evidence consistent with their hypothesis that the bacterial symbiont Spiroplasma compete with spermatozoa stored in female tsetse fly

spermathecae for acylcarnitines produced by the host female.

Most of the experimental work was completed in laboratory reared Glossina fuscipes

fuscipes (G.) using the bacterium Spiroplasma glossinidia (sG.). The symbiont sG. is

maternally transmitted, infecting tsetse gut, reproductive tracts, and hemolymph. sG.

infected tsetse are more resistant to trypanosome infection and have reduced fecundity

relative to uninfected individuals. Some work was also completed in field collected G.,

and some work was completed in laboratory reared Glossina mortisans mortisans because

it does not contain Spiroplasma.

I have a few comments/questions that will help improve the manuscript.

1) Include Table S1 in the manuscript. For those not familiar with the naming of acylcarnitines (probably most readers of this manuscript), the presence of the table in the main text would be very helpful.

*As the reviewer suggests, we have moved S1 Table into the main manuscript (it is now Table 1).*

2) Line 114: Define GC – what is this??

*GC, gonotrophic cycle, which is first defined in line 75 of the revised manuscript.*

3) Line 177: FB = Fat Body. It seems unnecessary to abbreviate throughout.

*We have removed the ‘FB’ abbreviation throughout the revised manuscript so that it now reads ‘fat body’.*

4) Line 199: “[shown here…” Where?? It was not clear where in the current study this is

shown.

*We have clarified this in the revised manuscript (line 208) by stating ‘[shown here in tsetse (S2C Fig.), and in reference…’. Specifically, we have added reference to the supplemental figure that shows this data).*

5) Lines 188-214 – I got really quite confused understanding where expression of acylcarnitines is up and where it is down, it seems there are differences in different tissues, but keeping this straight was impossible – a figure or table summarizing these patters would be very helpful.

*As the reviewer has suggested, we have made a supplemental table (S1 Table) that summarizes which acylcarnitines are more and less abundant in each tissue that we evaluated. We have referenced S1 Table in lines 231, 367, and 386 of the revised manuscript where we describe the results of each specific lipidomics data set.*

6) Line 240: “imbalance”– is there a different word that could be used here? How do you know that the different profile = an imbalance?

*As the reviewer rightfully suggests, we have removed the word ‘imbalance’ from the revised manuscript. This sentence (starting on line 257 in the revised manuscript) now reads as follows: ‘Taken together our untargeted (fat body lipidome) and the targeted (circulating hemolymphatic acylcarnitines) lipidomics analyses revealed that sGff infection induces a change in acylcarnitine homeostasis in mated Gff females.’*

7) Line 325: “to provide” seems to be overly confident language … it seems that the authors are speculating that this is the reason.

*The reviewer is correct that we are speculating that mating induces the female’s spermatheca to ramp up energy production via the carnitine shuttle to provide nutritional support to resident spermatozoa. As such, we state at the beginning of this sentence (line 350 in the revised manuscript) that ‘This outcome suggests that…’.We feel that the word ‘suggests’ implies that we do not have experimental proof that this is what’s happening and we are thus only speculating.*

8) Figure 4, panel E (lines 389-390) – why is it only the section marked by the dotted line and not the tissue around the whole spermathecae – there does not seem to be a difference in the tissue in the marked section of the tissue versus the whole yellowish halo.

*The reviewer makes a good point here, and as such in the revised manuscript we have circled all of the glandular cells that surround the left spermatheca in Fig. 4, panel E. We have also changed the corresponding figure legend (line 419 in the revised manuscript) to reflect this change.*

9) Line 416; “the enzyme”– what enzyme?

*To make this more clear we have replaced ‘the enzyme’’ with ‘CPT1’ (line 446 in the revised manuscript).*

10) Line 605: replace describing with described.

*We have made this change (line 667 in the revised manuscript).*

11) Figure 5 – I did not find this figure to be at all helpful, it made no sense to me. It would be great to have a figure that does clearly explain the findings and the model of regulation/competition proposed by the authors, but this figure it is not it.

*As the reviewer suggested we have changed Fig. 5 to simplify it and make it more useful to the reader. To do so we:*

*1) Removed the right side portion of the figure that contained information about Glossina morsitans morsitans (Gmm, which does not house Spiroplasma) so that the revised figure now focuses specifically on the interaction between Spiroplasma (sGff), Glossina fuscipes fuscipes (Gff), and acylcarnitines. We have also changed the figure legend (line 507 in the revised manuscript) to more accurately capture the simplified content of the revised figure. The revised figure now captures the main finding of the paper, which is the competition between the bacterium and fly for acylcarnitines, and how this competition impacts stored spermatozoa.*

*2) Removed the following sentence from the first paragraph of the discussion (lines 464-466 of the original manuscript), which corresponds to the removed Gmm portion of the figure: “Mating upregulates spermathecal production of acylcarnitines, which implies that these lipids are essential for the maintenance of stored spermatozoa and thus fly fecundity.” We moved this summary sentence (and made subtle changes to it) to the Results where we detail the changes in spermathecal lipid production following mating (lines 386-388 of the revised manuscript).*

*3) Although we removed the Gmm component from Fig. 5, we feel that a summary/model of the Gmm-related findings is still important. Thus, we have made that material into a separate supplemental figure (S10 Fig.) that we reference in the results after we summarize the relevant findings (line 480 of the revised manuscript).*

12) General comment on all figures, to work to increase the labelling on the figures to reduce the reader’s dependence on the figure legends. Doing this will increase the readability and accessibility of the manuscript.

*We feel that the figures are currently labelled in such a way that they reduce the reader’s dependence on the figure legends as much as can be achieved.*

**Reviewer #3**:

The manuscript titled “Endosymbiont hijacking of acylcarnitines regulates insect vector fecundity by suppressing the viability of stored sperm” describes a solid study investigating how the parasitic bacteria Spiroplasmsa glossinidia (sGff) impacts the viability of tsetse fly sperm stored in the female spermathecae (sperm storing organ). The authors show that sGff impacts motility in sperm stored in the female spermathecae, but not the male testes and use RNAi and gene expression analysis to provide mechanistic evidence that the impact on spermathecae stored sperm is mediated by fly-produced acylcarnitines. Overall, this is a clean study that presents an advancement in our understanding how Spiroplasma impacts tsetse fly fecundity. I have a few minor suggestions and comments that should be considered to improve the manuscript.

Suggestions and comments

Remove speculative statements beginning with “likely,” such as “likely other tsetse species as well” (Line 153), from the Results section.

*As the reviewer suggests, we have removed ‘(and likely other tsetse species as well)’ from the manuscript (line 157 of the revised manuscript). We also removed some other statements that include the word ‘likely’ where we feel that our data (or published data) validate the statement. However, we have left some speculative statements, mainly in the Discussion, because they are supported by the data but important for the context of the study.*

Lines 186–188: The statement “spermatozoa stored in the spermathecae of sGff+ females exhibit a motility defect” should be revised. Because sperm in male testes are inherently less active, and sperm are more active in spermathecae regardless of female infection status, it is not possible to distinguish a motility defect from an activation defect. The phrasing should remain agnostic to mechanism.

*We thank the reviewer for this comment, and have changed the statement in the revised manuscript (starting on line 198) to read as follows: ‘spermatozoa stored in the spermathecae of sGff^+^ females are less motile than are their counterparts that reside in the spermathecae of sGff^-^ females.’*

Lines 188–190: The claim that “mated Gff females that housed the bacterium presented with a relatively increased abundance of acylcarnitines in their FB” requires a reference (either to a publication or figure). The same applies to Lines 243–244: “We observed that the FB of mated sGff+ females produces more acylcarnitines than does that of their sGff– counterparts.”
*As the reviewer requests we have added ‘(S2C Fig.)’ as reference to the results that support the statements indicated above.*

What is the baseline lipidome of unmated females with and without Spiroplasma? If infection already alters lipid profiles before mating, this could affect interpretation of how sGff influences lipids in mated females.

*The reviewer raises a good point here. However, we did not quantify the baseline lipidome in virgin sGff^+^ and sGff^-^ females for the following reason. This study focuses on how sGff and stored (post-mating) spermatozoa compete for acylcarnitines. Thus, while we agree with the reviewer that sGff infection status may impact the lipidome in virgin females (and this is something we should investigate in future studies), our objective herein was to quantify the lipidome after mating in order to determine how its composition at that time relates to the stored spermatozoa motility phenotypes we observed sGff^+^ and sGff^-^ females.*

Where multiple t-tests were performed (e.g., Line 225), please clarify whether a multiple-testing correction was applied.
*We thank the reviewer for pointing out that we originally failed to add this information. Our multiple t-tests were subjected to false discovery rate (FDR) corrections, and this is now indicated where appropriate in the revised manuscript.*

The acronyms “sGff” and “Gff” are very similar and sometimes confusing. Consider using clearer or more distinct identifiers, as the similarity occasionally obscures meaning.

*We agree with the reviewer that these acronyms can be confused. However, the tsetse fly-Spiroplasma research community has designated the bacterium as ‘sGff’. Although this designation is informal, it appears in other publications, and for the sake of consistency, we would prefer to leave it as is.*

Line 245: The statement “This finding suggests that both hemolymph-borne and sGff localized within fly tissues may be metabolizing acylcarnitines” is not directly supported by the data. If retained, the reasoning should be explicitly stated; otherwise, it should be removed.

*As the reviewer suggests we have changed this sentence in the revised manuscript (starting on line 267) to read as follows: This finding suggests that hemolymph-borne sGff may be metabolizing acylcarnitines.*

Several parenthetical asides are too informal for a manuscript, such as Line 266: “[amongst other things, see Materials and Methods…].” These should be rephrased or omitted.

*As the reviewer suggested we have removed ‘amongst other things and other parenthetic asides from the revised manuscript (line 294).*

The manuscript alternates between metabolite measurements in hemolymph and gene expression perturbations in spermathecae. Although this design is reasonable, the authors should acknowledge that these are distinct tissues and that conclusions about their interaction are inferred. Because Gff can inhabit the gut, this could also influence hemolymph lipid changes. Likewise, etomoxir treatment affects multiple tissues, so a more holistic discussion of its systemic effects—both within and outside the spermathecae—would strengthen the interpretation.

*In response to this insightful suggestion, we have added two sentences (starting on line 502 of the revised manuscript) that highlight the systemic nature of sGff infection and how this situation impacts acylcarnitine homeostasis and the motility of stored spermatozoa. These sentences state specifically: ‘Additionally, although not experimentally demonstrated in this study, sGff that reside in tsetse’s gut presumably also utilize acylcarnitines. Such a cumulative impact on distinct fly tissues insinuates that the bacterium exerts a systemic effect on acylcarnitine homeostasis that impacts their availability locally in the mated female’s spermatheca.*

The abbreviation “GC” should be reintroduced when it reappears in the Results, as it was easy to forget by that point.

*We have redefined the abbreviation ‘GC’ the first time it is mentioned in the Results section of the revised manuscript (line 462).*

The reduced Sodalis loads observed in dsCPT1 flies merit more discussion. Although the authors state that Sodalis “has no known function related to tsetse reproduction,” absence of evidence is not evidence of absence. This finding should be interpreted rather than dismissed.

*The reviewer makes a good point here. We have thus edited the sentence that mentions the detrimental effect of cpt1 knockdown on Sodalis to read as follows (lines 477-480 in the revised manuscript): ‘Conversely, this treatment did significantly repress Sodalis [this bacterium has no known function related to tsetse reproduction (15, 57)], which, like sGff, systemically infects tsetse and may also consume acylcarnitines as an energy source’. This revised sentence highlights our interpretation of this outcome, which is that Sodalis may also consume acylcarnitines, and the bacterium’s density thus decreases when cpt1 transcript abundance is experimentally reduced via RNAi.*

It would be helpful to mention earlier that sGff cannot synthesize lipids de novo, as this is an important aspect of its metabolism.

*As the reviewer has suggested we have added this information to the introduction of the revised manuscript (line 120)*.

An additional consideration is that sGff-infected females might need to mate more frequently to maintain fecundity, potentially providing a transmission advantage to sGff. This possibility is worth noting.

*The reviewer makes another interesting point here. However, we respectfully decline to make corresponding changes to the manuscript for the following reason. Regardless of how many times sGff^+^ females mate (there is evidence of polyandry in wild flies), stored spermatozoa (originating from one or multiple mates) would have to compete with the bacterium for acylcarnitines and thus exhibit reduced motility. sGff would have a transmission advantage if infected females produced offspring more frequently. However, this does not happen, as infected females exhibit a longer gonotrophic cycle and thus produce fewer offspring than do uninfected females.*

Lines 526–527: The statement “we observed glandular cells associated with the outer perimeter of tsetse’s spermathecal reservoir” should clarify whether this is a novel observation or one previously reported.

*We report in the manuscript (sentence starting on line 584 in the revised manuscript) that glandular cells have previously been observed in tsetse’s spermatheca (references 73 and 74), although their function is unknown (and still technically unknown, although we state in our discussion that our data imply these cells produce acylcarnitines because they are the one metabolically active ones in this tissue).*

Finally, please remove asterisks from lipidome panels where compounds only trend toward a difference but are not statistically significant. Because asterisks conventionally denote significance, an alternate marking would be more appropriate.

*As the reviewer suggested we have removed the blue asterisks from the lipidome panels and replaced them with blue hashtags.*
